# Application of frass from black soldier fly larvae treatment of cattle dung in pulp and papermaking

**DOI:** 10.1038/s41598-024-53496-0

**Published:** 2024-02-05

**Authors:** Hao-Chen Sun, Yu-Hsun Lai, Jiann-Gwo Shyu, Yuan-Shing Perng

**Affiliations:** 1 Department of Forestry, National Chung Hsing University, Taichung, 40227 Taiwan, ROC; 2 Forest Products Utilization Division, Taiwan Forestry Research Institute, Ministry of Agriculture, Taipei, 100051 Taiwan, ROC

**Keywords:** Environmental sciences, Materials science

## Abstract

Cattle dung treatments in Taiwan have developed a process called Black soldier fly larvae (BSFL) treatment, which can digest cow dung and generate the frass (larvae drops), the residue fiber in cow dung. This study aims to assess frass for its potential in pulp and papermaking, considering its chemical compositions, appearance, and fiber morphology, and also evaluate its suitability for pulping by soda method to create added value. The frass exhibits favorable material properties for pulping and papermaking, including a high holocellulose (67.37%) and α-cellulose (48.00%) content, along with a lower ash content (4.61%); the microstructure and surface mesoporous pores benefit for pulping; and the nonwood-fiber-like fiber morphology. The pulping experiment shows that 7% NaOH and 75 min of pulping conditions result in proper disintegration of fiber, and the highest accepts ratio (34.06%). The NaOH causes fiber disintegration during pulping, resulting in a higher strength property of the handsheet. The frass pulp blended with TOCC can achieve the ring crush index standards required for cardboard products. In summary, the frass from BSFL treatment of cattle dung can be utilized in pulp and papermaking to enhance circular utilization value.

## Introduction

According to livestock and poultry statistical survey results (Q2, 2023)^1^ from Taiwan's Ministry of Agriculture, 160,014 cattle were raised during the second quarter of 2023, representing 8% of the country's total livestock production. Each cow produces approximately 30 kg of dung per day^2^, resulting in a daily treatment requirement of 4,800 tons of cattle dung in Taiwan. With the growing focus on circular agricultural waste utilization in recent years^3,4^, the value-added utilization of cattle dung treatment has become an important concern.

The Husbandry Excrement Resources Web^5^ presents information about resource-based applications and treatment of cattle dung, which indicates that the main method of cattle treatment is anaerobic fermentation. The anaerobic fermentation of cattle dung produces biogas to generate electricity. In the meantime, the remaining slurry and residue solid are returned to agriculture as fertilizer. Not only anaerobic fermentation but also black soldier fly (*Hermetia illucens*) larvae (BSFL) have been utilized to digest cattle dung in some studies^6,7^. After the cattle dung is treated by BSFL, the larvae can be applied in protein generation^8^, and the residue matter is collectively referred to as frass. The frass has different characteristics according to the raw organic matter, thus, the frass can be utilized in various applications, such as fertilizer, energy, material, feed, and other directions^9^. The BSFL treatment of cattle dung is applied in Taiwan Livestock Research Institute, which produces the frass that is similar to undigested cattle dung fiber (CDF). According to the information provided by Taiwan Livestock Research Institute, the BSFL treatment can transfer the cattle dung with a 30% yield to the frass. However, the institute also indicates that frass utilization still needs to be created to improve the development of BSFL treatment on cattle dung.

The undigested fiber content is approximately 40 ~ 50% in cattle dung^10^. During the digestion process of cattle, the forage grass and feed are damaged by physical (chewing) and microbial enzyme treatment, cutting off fiber and degrading components such as hemicellulose and pectin, so CDF is different from the state of original forage and feed. Simultaneously, the degradation of hemicellulose and pectin might cause the CDF to have a higher cellulose content in reasonable inference^11,12^. CDF has potential in fiber application development and can be utilized in value-added fiber products, such as animal bedding, particleboard, wood-plastic composites, plant pot, and fuel production^13^. Based on the above, the CDF-like frass from cattle dung BSFL treatment might have potential in the development of value-added fiber applications.

The pulp and papermaking industry is one of the largest global industries, consuming a significant amount of fiber feedstock. The demand for paper and paperboard has been steadily increasing each year^14^, consequently driving the demand for fiber feedstocks. Therefore, the pulp and papermaking industry must expand more fiber sources. CDF applied in pulp and paper has been researched in some studies. Vishnuvarthana et al.^15^ utilized a combination of banana fiber and CDF to create a handsheet, aiming to assess its potential for eco-friendly packaging applications. Fasake and Dashora^16^ analyzed the cellulose content, appearance structure, and chemical composition of CDF to evaluate the feasibility of CDF in biofiller applications. in the pulp and papermaking industry. Furthermore, Fasake et al.^17^ subjected the CDF to hot water treatment at temperatures ranging from 120°C to 180°C and analyzed the yield, chemical compositions, and physical changes. Yang et al.^18^ treated CDF with hot water, 5 wt% H_2_O_2_, 5 wt% NaOH, and 5 wt% KOH at 90 °C for 2 h each to extract cellulose and create the handsheets for property analysis. The results in the above works of literature both show the utilization potential and feasibility of CDF in fiber utilization, especially in papermaking.

However, the optimization of the actual pulping process, including the methods (kraft, soda) and the conditions (time, temperature, chemical dosage), has not been determined^19^. Therefore, it is necessary to determine the optimized conditions of CDF pulping in traditional methods, especially the frass pulping process, to figure out the pulping properties of traditional methods, then start to develop other novel pulping processes or conditions, which can probably improve the circular utilization of cattle dung with BSFL treatment. Among the traditional pulping processes, chemical pulping, especially soda method, has been industrially adopted in pulping of agricultural surplus^20–22^. Consequently, this research opts for the soda methods to assess the pulping properties of the frass.

The study aims to discuss the pulp and papermaking potential of the frass from cattle dung after BSFL treatment by analyzing the chemical compositions, appearance, and fiber morphology to assess the pulping and papermaking potentials of the frass and optimizing the soda pulping process through the experimental design of 2^2^ factorial analyses to evaluate the impact of digestion time and NaOH dosage on pulping properties, including pulping yield, freeness, and handsheet properties. After the determination of the pulping potentials and properties, the highest yield sample of the frass pulp is blended with TOCC, and a handsheet is created to evaluate the feasibility of the application for industrial paper. The study hopes that the application assessment of the frass can enhance the value-added potential of cattle dung processed by BSFL.

## Experiment

### Material

**Figure 1:**
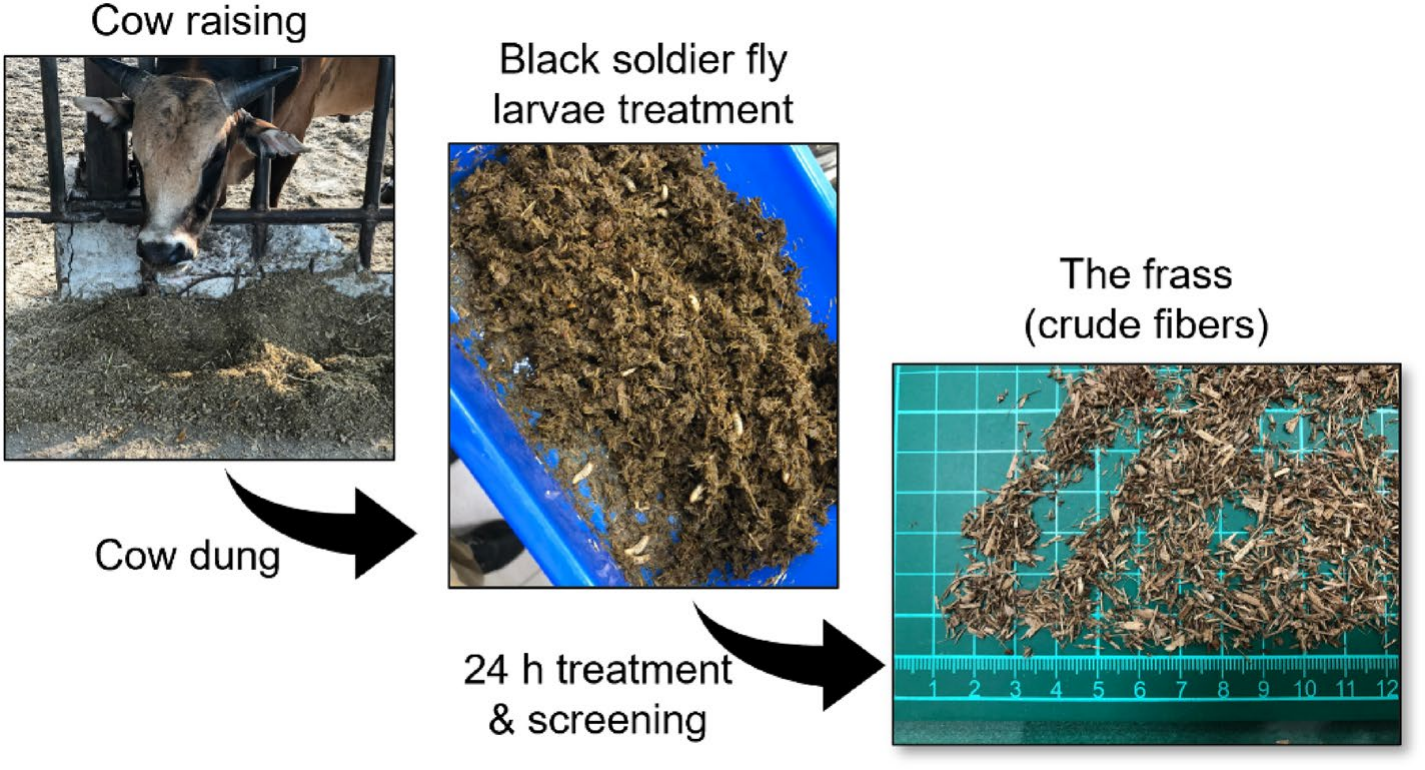
The process of treating cow dung with black soldier fly larvae.

The frass, which is the residue from BSFL treatment of cattle dung, was collected on November 18, 2022, and provided by Taiwan Livestock Research Institute, Ministry of Agriculture, Tainan, Taiwan. According to the information from Taiwan Livestock Research Institute, the process of BSFL treatment is shown in Fig. 1. The cattle dung is collected by the operators and then put into the BSFL treatment for 24 h. After BSFL treatment, the frass is screened to remove the larvae and dried by the sun for two days. The air-dried solid content of the frass is 84.63%. Crude-fiber-like frass (Fig. 1) is stored in a plastic bag and prepared for experiments. The TOCC and JOCC pulps were provided by Houli Mill, CHENG LOONG Co., Ltd. (Taichung, Taiwan), with freeness of 385 mL and 405 mL, and pulp consistency of 39.23% and 37.66%, respectively.

### Chemicals

The chemicals used in this study are as follows: 95% alcohol, NaOH, and H_2_O_2_ of Extra pure grade are provided by KATAYAMA PURE CHEMICAL Co., Ltd., Changhua, Taiwan. Toluene, acetone, hydrochloric acid, glacial acetic acid, and sulfuric acid of reagent chemical grade are provided by DEJUNG, Korea. Sodium chlorite of Extra pure grade is provided by ECHO CHEMICAL Co. Ltd., Taichung, Taiwan.

### Method

**Figure 2:**
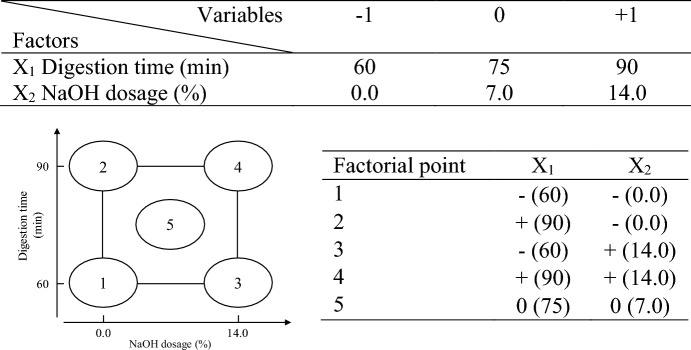
Factorial experimental design of CDF soda pulping.

The constant conditions for the frass pulping experiment included a digestion temperature of 170℃, which is according to the traditional chemical pulping temperature^23^, a solid-to-liquid ratio of 1:8, which is set according to the chemical pulping conditions of Napier grass, one of cattle feeds^24^, a heating rate of 1.5℃/min, and a material weight of 500 g (oven-dried weight, odw). The factorial experimental design in Fig. 2 includes two variables. The digestion time (X_1_) is set at a high level (+ 1) of 90 min and a low level (− 1) of 60 min, which is also set according to the traditional chemical pulping temperature^23^, while the NaOH dosage (X_2_) is set at a high level (+ 1) of 14% and a low level (-1) of 0%, which the high level is set according to pulping conditions of Napier grass and the low level is set according to Fasake et al.^17^. These conditions result in four factorial points, each representing one experiment. To assess the significance of the impact of these two variables, the study conducts a double experiment at the midpoint (0) with a digestion time of 75 min and a NaOH dosage of 7% to calculate the assessed value.

During the pulping process, first, the frass is soaked until completely moistened by additional water, calculated by the solid-to-liquid ratio, and then mixed with the calculated NaOH (g) and placed into the rotary digester (Model 8060, LESSON INDUSTRIAL Co., Ltd., New Taipei, Taiwan) to start pulping. After digestion and cooling, the pulp was washed under tap water using a 200 mesh sieve until the filtered water became colorless. The mesh size was selected according to the Fines definition; thus, matters passing 200 mesh were referred to as Fines^25,26^. When the pulping process is finished, the pulp is placed into a plastic bag in preparation for analysis.

### Material properties

#### Chemical composition

The frass was pulverized by a high-speed pulverizing machine (RT-02B, RONG TSONG Precision Technology Co., Ltd., Taichung, Taiwan), and then the particles were collected between 40 and 60 mesh for analysis, including ash (TAPPI T 211 om-22), alcohol-benzene extractives (TAPPI T 204 cm-17), holocellulose (ASTM D1104-56), acid-insoluble lignin (TAPPI T 222 om-21), and α-cellulose (TAPPI T 203 cm-22). Each analysis was repeated twice to calculate the average and standard deviation.

#### SEM–EDS

The frass appearance and the handsheet of each factorial point were observed by scanning electron microscopy (SEM, ZEISS ULTRA PLUS, ZEISS, Germany) under an operating voltage of 15 kV. In the meantime, the surface elemental composition of the frass was assessed through energy-dispersive X-ray spectroscopy (EDS, OXFORD X-Max 50 mm^2^, Oxford Instruments, UK).

#### Fiber morphology

The frass is disintegrated at 40℃ for 7 days by a digestion solution comprising 1 part hydrogen peroxide, 5 parts glacial acetic acid, and 4 parts distilled water according to Franklin’s method ^\*MERGEFORMAT^^27^ The disintegrated fiber is measured by a fiber image analyzer (FS5, Valmet Co., Ltd., Finland), and the fiber length (mm), fiber width (μm), cell wall thickness (μm), coarseness (mg/m), and L/D value (length to width ratio) are recorded. All the samples were analyzed 6 times to record the average and standard deviation.

### Pulp properties

#### Pulping yield

After pulping, the pulp sample is screened by a flat screen (Model 302, LESSON INDUSTRIAL Co. Ltd., New Taipei, Taiwan) with a slot width of 0.25 mm according to TAPPI UM 242. The material unable to pass through the 0.25 mm sieve is termed Shives, while that passing through and collected by a 200 mesh sieve is referred to as Accepts. The lost material is collectively termed Fines^25,26^. After screening, Collective Shives and Accepts measured the total oven-dried weight and determined the Shives, Accepts, and Fines ratios using Eqs. (1), (2), and (3), respectively.

1 $$\mathrm{Shives \;ratio }\left(\mathrm{\%}\right)=\frac{Shives \;oven \;dried \;weight (g)}{Raw \;material \;oven \;dried \;weight \;(g)}\times 100$$

2 $$\mathrm{Accepts \;ratio }\left(\mathrm{\%}\right)=\frac{Accepts \;oven \;dried \;weight (g)}{Raw \;material \;oven \;dried \;weight \;(g)}\times 100$$

3 $$\mathrm{Fines \;ratio }\left(\mathrm{\%}\right)=100-\mathrm{Shives \;ratio }\left(\mathrm{\%}\right)-\mathrm{Accepts \;ratio }(\mathrm{\%})$$

#### Pulp freeness

Pulp freeness was measured by a Canadian standard freeness (CSF) tester (Model 305, LESSON INDUSTRIAL Co., Ltd., New Taipei, Taiwan) according to TAPPI T 227 om-17. Each sample is tested twice, and the average result is recorded.

#### Accepted fiber appearance

The accepted fiber appearance of each factorial point was observed by a optical microscopy (ECLIPSE E100, Nikon, Japan). Before the observation, the pulp concentration was adjusted to 0.4% to disperse the fibers for specimen preparation. During the observation, images were captured by a microscope camera (HDC588, MicroTech, Canada) with MicroCam V5 software (M&T OPTICS Co., Ltd., Taipei).

#### Handsheet properties

The accepted fiber pulp of each factorial point in Fig. 2 is made the handsheet to analyze the impact of pulping conditions on handsheet properties. In the meantime, the sample with the highest pulping yield is blended with TOCC at frass pulp to TOCC ratios of 100/0, 75/25, 50/50, 25/75 and 0/100. After blending, the freeness and handsheeting properties of the mixed pulp are analyzed to evaluate the feasibility of frass pulp application in cardboard production. The handsheet was made according to TAPPI T 205 sp-12 with the grammage setting at 100 g/m^2^ to simulate the cardboard grammage. Before testing, the handsheet was conditioned under 50.0% ± 2.0% RH and 23.0 ± 1.0 °C for at least 4 h according to TAPPI T 402 sp-13. The grammage and thickness of the handsheet were measured according to TAPPI T 410 om-08 and T 411 om-21, respectively, to calculate the bulk value. Six handsheets with similar grammage and intact appearance are selected to test the strength properties. The testing items are mainly the paper properties specified by Taiwan CNS standards for cardboard paper, including the tensile index (TAPPI T 494 om-01), burst index (TAPPI T 403 om-15), and ring crush index (TAPPI T 818 cm-18). The experimental results are recorded by calculating the average value and standard deviation of 6 test data.

### Statistical analysis

The factorial design analysis followed the guidelines outlined in 'Design and Analysis of Experiments 7/e' by Montgomery^\*MERGEFORMAT^^28^, using Microsoft Excel (Microsoft, USA) for analysis. The handsheet properties of the frass pulp blended into TOCC were analyzed by IBM SPSS Statistics 20 (IBM, USA) for one-way analysis of variance (ANOVA). The Tukey honestly significant difference (HSD) test was employed for multiple comparisons, employing a 95% confidence interval. A *p* value < 0.05 indicated statistical significance.

## Results and discussion

### Material properties

#### Chemical compositions

The chemical compositions of the frass are shown in Table 1, with an ash content of 4.61%, a toluene-alcohol extractives content of 3.89%, a holocellulose content of 67.37%, an acid-insoluble lignin content of 17.01%, and an α-cellulose content of 48.00%. The frass similar to the CDF, so the result is compared with the CDF researched by Fasake and Dashora^16^ and Yang et al.^18^. In comparison, the toluene-alcohol extractives, holocellulose, acid-insoluble lignin and α-cellulose content of frass are relatively higher, and the ash content is lower, which the result of higher holocellulose shows the greater potential of fiber application. The study infers that the BSFL treatment can digest most of the organic matter in cattle dung and transfer it into insect protein, which results in the residue matter being undigested fibers that BSFL cannot digest. In this case, to compare with the wash treatment of cattle dung in Fasake and Dashora ^\*MERGEFORMAT^^16^, the crude fiber obtained from BSFL treatment might have fewer impurities, so the process leads to crude fibers (frass) with higher applied potential^29^.

**Table 1:** Chemical compositions of the frass and CDF.

Material		Ash (%)	Toluene-alcohol extractives (%)	Holocellulose (%)	Acid insoluble lignin (%)	α-cellulose (%)	References
The frass		4.61 ± 0.08	3.89 ± 0.31	67.37 ± 0.05	17.01 ± 1.21	48.00 ± 0.29	The study
CDF	1	7.06 ± 0.70	1.64 ± 0.08	54.8 ± 0.94	12.96 ± 0.69	31.34 ± 0.91	Fasake et al.^17^
	2	5.49 ± 0.08	1.35 ± 0.07	45.01 ± 1.11	12.06 ± 0.74	29.94 ± 1.65	
	3	4.09 ± 0.57	2.00 ± 0.12	52.22 ± 0.58	11.97 ± 0.71	31.19 ± 0.82	
CDF		5.79	2.28	69.86	21.31	41.6	Yang et al.^18^

*CDF* Cattle dung fiber.

The comparison of the frass and the other fiber materials' chemical compositions is shown in Table 2. The nonwood fibers (*Pennisetum alopecuroides*^30^, cornstalk^31^, rice straw^32^ and wheat straw^17^) in Table 2 have been recorded in the application of pulping and cattle raising. According to the study's results, it was found that the holocellulose and α-cellulose content in the frass is higher, while the acid-insoluble lignin content is lower. This indicates that despite being grass digested by cattle, the chemical composition of frass is still similar to that of other nonwood fibers, and it even contains a higher cellulose content. The comparison between the frass and traditional pulping raw materials (softwood and hardwood)^33^ shows similar holocellulose and α-cellulose contents; in addition, the lignin content is lower. In summary, the chemical composition of the frass has a higher cellulose content and lower lignin content in comparison to other fiber materials, which shows the great potential of application in pulp and papermaking.

**Table 2:** Chemical compositions of the frass and other fiber materials.

Material	Ash (%)	Toluene-Alcohol extractives (%)	Holocellulose (%)	Lignin (%)	α-cellulose (%)	References
The frass	4.61 ± 0.08	3.89 ± 0.31	67.37 ± 0.05	17.01 ± 1.21	48.00 ± 0.29	This study
Pennisetum Alopecuroides	6.76	–	69.5	19.66	40	Elmoudnia et al.^30^
Cornstalk	8.4	9.9	–	20.1	47.6	Hájková et al.^31^
Rice straw	15.8	1.7	59.0	21.0	31.1	Lai et al.^32^
Wheat straw	7.15 ± 0.77	3.07 ± 0.24	-	13.91 ± 0.52	39.36 ± 0.64	Fasake et al.^17^
Softwood	–	–	65–74	25–31	40–45	Gharehkhani et al.^33^
Hardwood	–	–	67–82	16–24	43–47	

#### Material appearance

**Figure 3:**
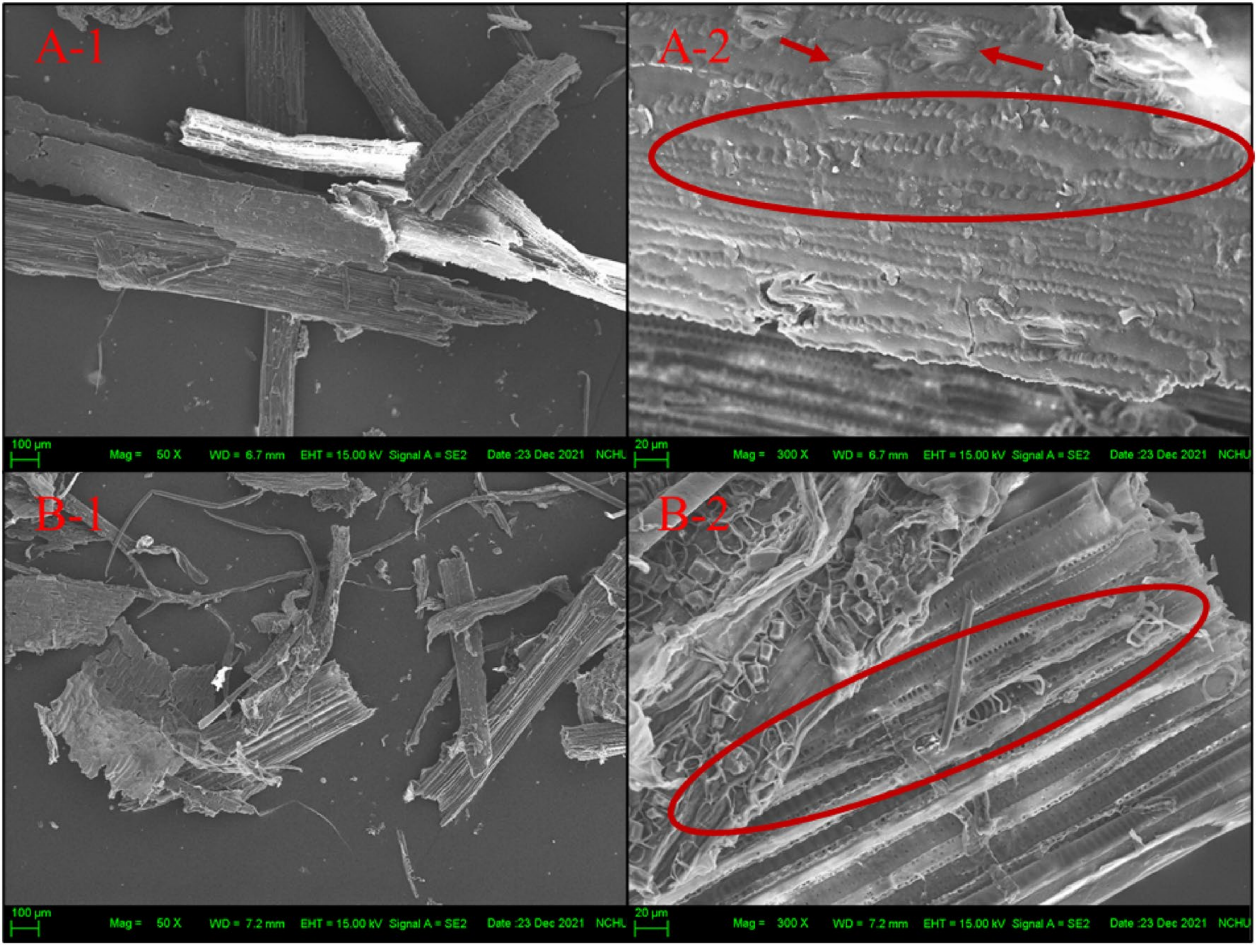
SEM images of the frass (**A**) sample 1 and (**B**) sample 2 (Codes 1 and 2 are associated with 50X and 300X magnification, respectively).

The frass is the fiber digested by cattle, so the study observes the frass by SEM to determine the status of the appearance. The SEM images are presented in Fig. 3. In Fig. 3(A-1) and (B-1), it is evident that the frass contains intricate and multiple crude fibers, a characteristic also observed in the CDF study by Yang et al.^18^. In Fig. 3(B-2), the fiber exhibits an exposed structure, with numerous small pores arranged continuously, as highlighted in the red circle. These small pores can be inferred to be “mesoporous pores," which can increase the roughness of the fiber surface and the hydrophilicity, which can improve the pulping efficiency, according to Yang et al.^18^. The fiber in Fig. 3(B-2) has particle-like matter on the surface, the larger particles pointed by red arrows are inferred as plant stomata^34^, and there are many arranged small particles surrounded by red circles that are similar to the surface trichomes of rice straw^35^. Based on the above, after the fiber is digested by cattle and through BSFL treatment, part of the surface structure of frass is destroyed, and part of the fiber still retains the original tissue and status of the plant. Although the frass fiber is varied and subjected to different degrees of digestion and degradation, the exposed structure and the mesoporous pores of the frass fiber are beneficial for pulping applications.

#### Surface elements

**Figure 4:**
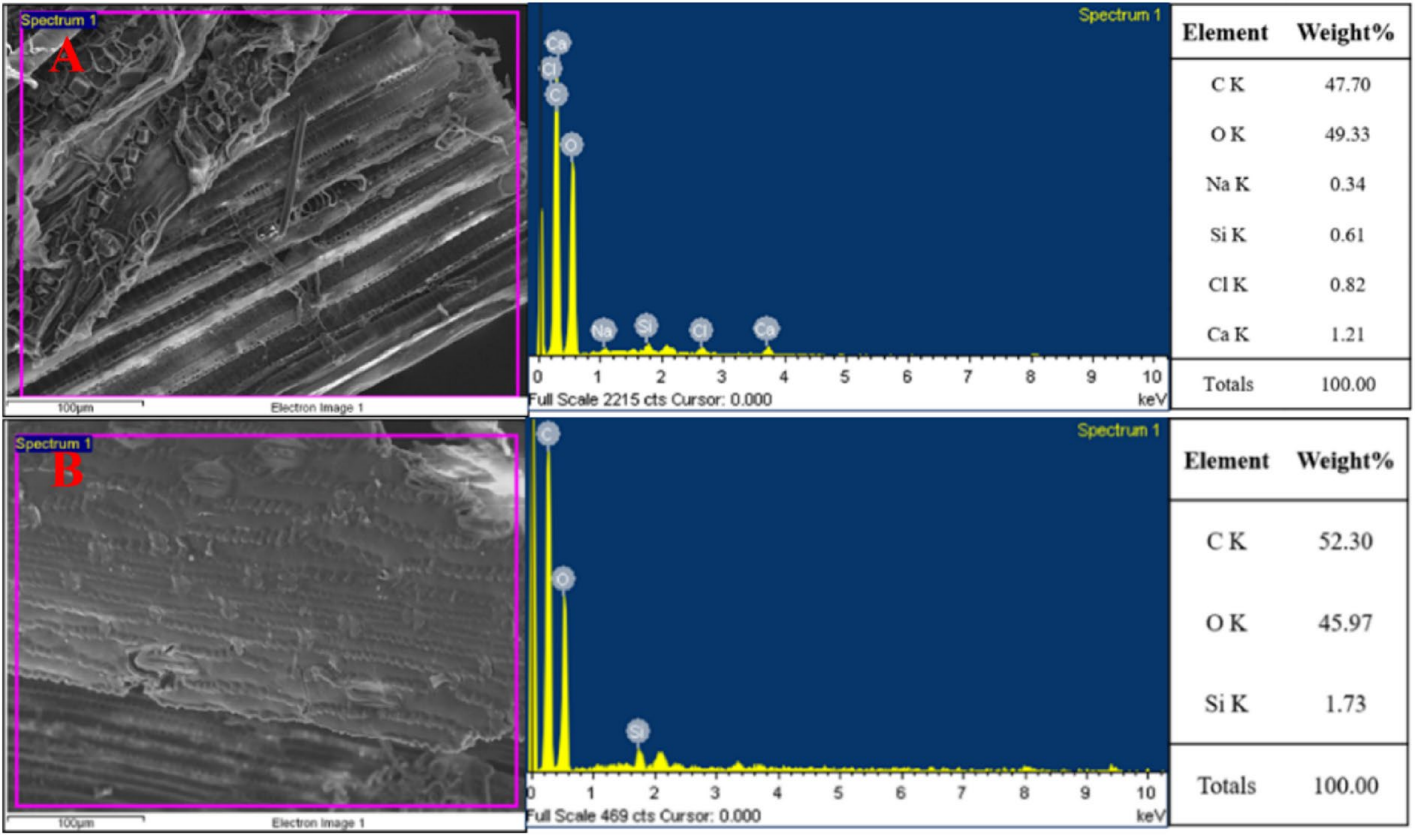
EDS analysis of the frass.

To further observe and analyze the trichome-like structures in Fig. 3(A-2), this study further conducted EDS analysis of the composition elements on the surface of the frass, as shown in Fig. 4. In the comparison of the two samples (Fig. 4A,B), it can be observed that the exposed structure exhibits more metal elements, such as Na and Ca. While the Si content in Fig. 4A is 0.61%, sample-2 in Fig. 4B shows a higher Si content (1.73%) on the surface. The study infers that the higher Si content in Fig. 4B might be due to the trichomes on the frass surface, which is similar to the matter containing SiO_2_ on the rice straw surface^35^. Yang et al.^18^ show a similar composition on CDF, which also has Si on the surface. The existence of Si in the residue fiber of cow dung might be due to the feed including corn, rice, and wheat straw, which both contain high Si^36–38^. If the high-content of Si appears in black liquor, it will become a problem during black liquor recovery^39,40^. However, the frass does not have as much ash content as other nonwoods, so the influence of Si in the ash on black liquor might be lighter.

#### Fiber morphology

The properties of papermaking applications are impacted by fiber morphology, so the study disintegrated the frass fibers according to Franklin’s method and measured the fiber morphology with afiber image analyzer (FS5) to evaluate the papermaking feasibility of the frass fibers. The results are shown in Table 3. The fiber morphology of the frass fiber includes a fiber length (Fl) of 0.788 mm, fiber width (Fw) of 21.46 μm, cell wall thickness (CWT) of 0.59 μm, coarseness of 0.039 mg/m, and a L/D value of 36.72. Compared with other materials, the Fl of the frass is similar to that of nonwood fibers (0.48~0.97 mm), especially rice straw (0.78 mm) in Ferdous et al.^41^. The Fw of the frass is wider than that of nonwood fibers (9.3~15.7 μm), similar to that of hardwood (22 μm), and narrower than that of softwood (40 μm). However, in the meantime, the CWT and the coarseness of the frass are lower than others, and it can be inferred that the cell wall density of the frass is lower. The L/D result shows that the L/D of frass is lower than that of other materials (57.32~104.30). Based on the above, it can be found that the frass fiber is similar to nonwood fibers, which might have apotential in fiber application developments. Although a lower L/D ratio of the frass, especially below 70, may lead to diminished physical strength properties during the papermaking process^42^, the CWT and coarseness of the frass are lower, which can produce a higher fiber contact area during paper formation^43,44^. The study infers that the fiber morphology of the frass has the potential for papermaking applications.

**Table 3:** Fiber morphology of the frass and other fiber materials.

Material	Fl (mm)	Fw (μm)	CWT (μm)	Coarseness (mg/m)	L/D	References
The frass	0.788 ± 0.005	21.46 ± 0.13	0.59 ± 0.03	0.039 ± 0.001	36.72 ± 0.15	The study
Rice straw	0.78	11.6	1.83	0.086	67.24	Ferdous et al.^41^
Wheat straw	0.97	9.3	1.99	0.095	104.30	
Corn stalk	0.90	15.7	1.78	0.092	57.32	
Pennisetum	0.41	–	–	–	–	El Omari et al.^42^
Birch	1.8	20–36	3–4	0.05–0.08	50–90	Smook^44^
Hardwood	2.0	22	–	–	90	
Soft wood	4.0	40	–	–	100	

*Fl* Fiber length, *Fw* Fiber width *CWT* Cell wall thickness *L/D* length/width ratio.

### Pulp properties

#### Pulping yield

**Figure 5:**
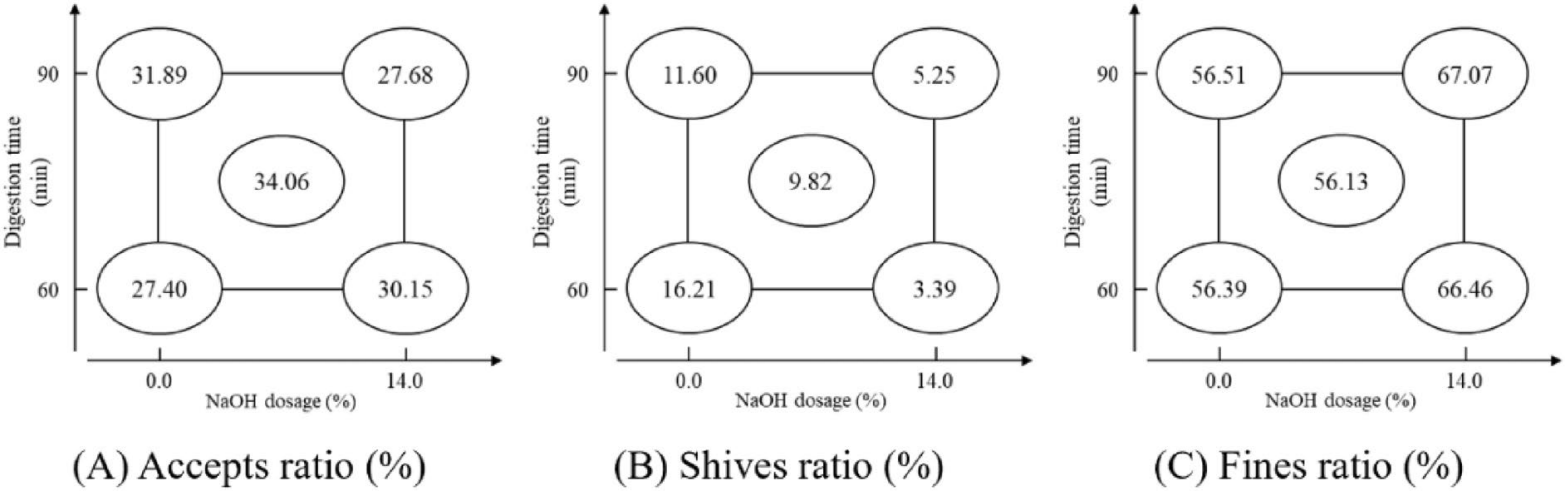
Factorial analysis charts of pulping yield.

After the material characteristic analysis, it can be confirmed that the frass has potential in pulp and papermaking, so the study tests the soda pulping properties of the frass with a 2^2^ factorial experimental design. The impact analysis of the pulping yield is shown in Table 4. The accepts ratio is significantly affected by the interaction (X_1_X_2_) between digestion time and NaOH dosage, and the impact value is -3.480. The shives ratio is significantly affected by NaOH dosage (X_2_) and the interaction effect (X_1_X_2_), with impact values of − 9.585 and 3.235, respectively, in which the absolute value comparison that X_2_ > X_1_X_2_ refers to more impact with NaOH dosage. The fines ratio is significantly affected by NaOH dosage (X_2_), with impact values of 10.315. The factorial analysis chart of pulping yield is shown in Fig. 5. The comprehensive comparison of Fig. 5A Accepts ratio, (B) Shives ratio & (C) Fines ratio and Table 4 shows that digestion time (X_1_) mainly affects the accepts and shives ratio. At 0% NaOH dosage, a longer digestion time can increase the accepts ratio, but with a 14% NaOH dosage, the accepted fiber will be over degraded, leading to a decreased accepts ratio. The NaOH dosage (X_2_) mainly affects the shives and fines ratio; the shives will have a higher disintegrated level with the increased NaOH dosage, and in the meantime, the fines will also increase. Due to the interaction between the two factors, the midpoint in the study reached the highest accepts ratio (34.06%). However, the highest accepts ratio is lower than the traditional chemical pulping yield of 40~55%^23^, which might be due to the higher fines generation during the frass pulping. Thus, the frass needs a proper digestion time and NaOH dosage during the pulping process to efficiently disintegrate the shives and reduce the accepted fiber degradation.

**Table 4:** Factorial analysis of pulping yield.

Factor	Accepts ratio (%)	Shives ratio (%)	Fines ratio (%)
Factor X_1_	1.010	− 1.375	0.365
Factor X_2_	− 0.730	− 9.585 *	10.315 *
X_1_X_2_ interaction	− 3.480 *	3.235 *	0.245
Assessed value	2.246	1.437	0.809
Pooled standard deviation (s)	0.177	0.113	0.064

*Means the factor impact is significant.

*Factor X*_*1*_ Digestion time, *Factor X*_*2*_ NaOH dosage.

#### Accepted fiber appearance

**Figure 6:**
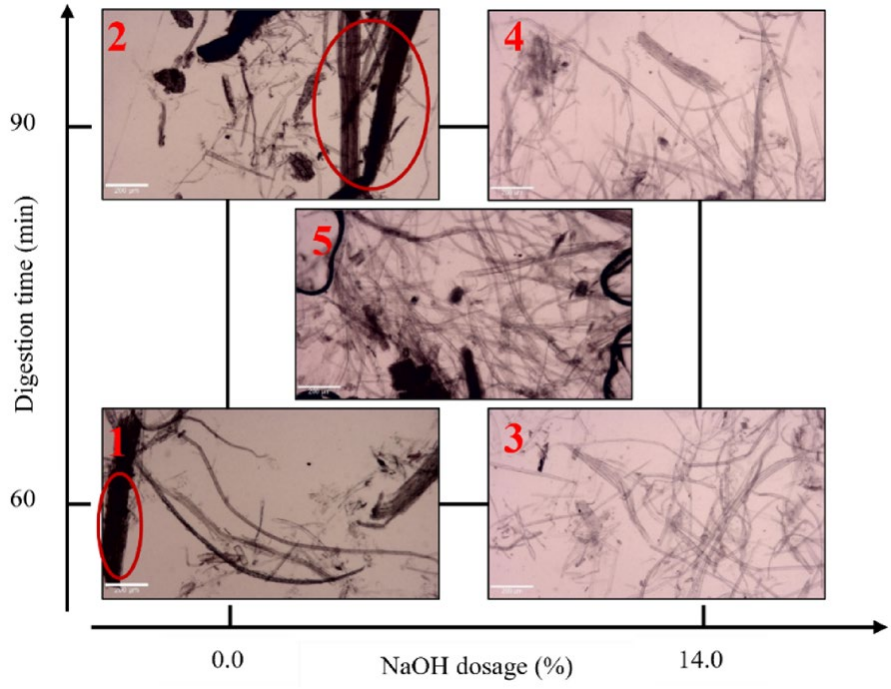
Optical microscope images of accepted fibers.

The accepted fibers are observed under the optical microscope, and the images taken at a magnification of 40X are shown in Fig. 6, with the images arranged in a factorial design chart. As the NaOH dosage varies, the differences at each factorial point become more significant. In contrast, the impact of digestion time is less noticeable. In Fig. 6, the factorial points 1 and 2 show the frass fiber that is not disintegrated, as highlighted in the red circle. When the NaOH dosage is higher (14%), as points 3 and 4, more single fibers appear, indicating a higher level of disintegration. The status of the midpoint is between points 1 and 2 and points 3 and 4. In the comprehensive discussion of fiber appearance and pulping yield, the study infers that the disintegrated level of the frass not only affects the appearance of fiber but also has an impact on pulping yield. The lower NaOH dosage with less disintegration of fiber causes a higher shives ratio, and the higher dosage causes more disintegrated fibers and fines, which are due to the NaOH impact on delignification and the peeling reaction during the soda pulping methods^45^. Based on the above, the study infers that the soda pulping of the frass needs a proper NaOH dosage (7%), which can optimize fiber disintegration and pulping yield.

#### Freeness

**Figure 7:**
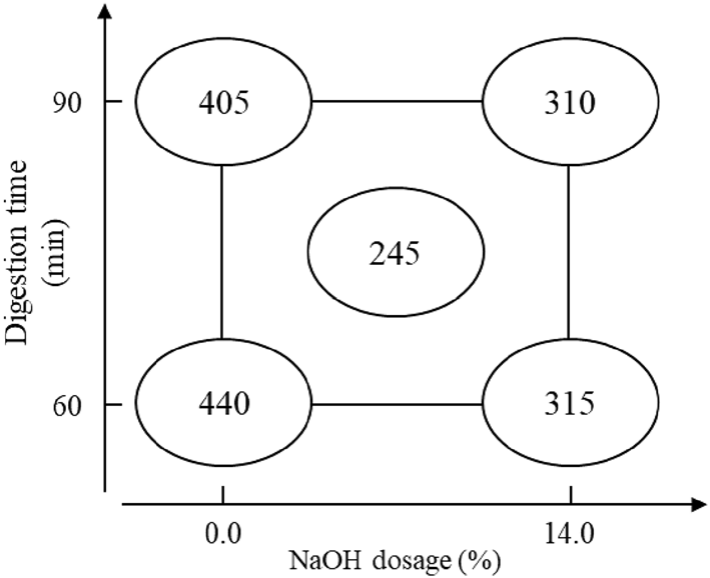
Factorial analysis chart of pulp freeness.

The freeness of the frass pulp is directly measured after screening, and the factorial analysis result is presented in Table 5. The NaOH dosage (X_2_) has a significant impact on freeness, with an impact value of -110.0. In Fig. 7, the factorial analysis chart shows that the freeness decreases as the NaOH dosage increases under constant digestion time. Hence, the NaOH dosage significantly influences the reduction in freeness during the frass soda pulping process. However, the lowest freeness occurs in the midpoint condition, and the study infers that the midpoint with the proper dosage causes the fiber to have appropriate disintegration, which has a higher accumulation density.

**Table 5:** Factorial analysis of pulp freeness.

Factor	CSF (mL)
X_1_	− 20.0
X_2_	− 110.0 *
X_1_X_2_ interaction	15.0
Assessed value	89.8
Pooled standard deviation (s)	7.1

*Means the factor impact is significan.

*Factor X*_*1*_ Digestion time, *Factor X*_*2*_ NaOH dosage.

### Handsheet properties

**Figure 8:**
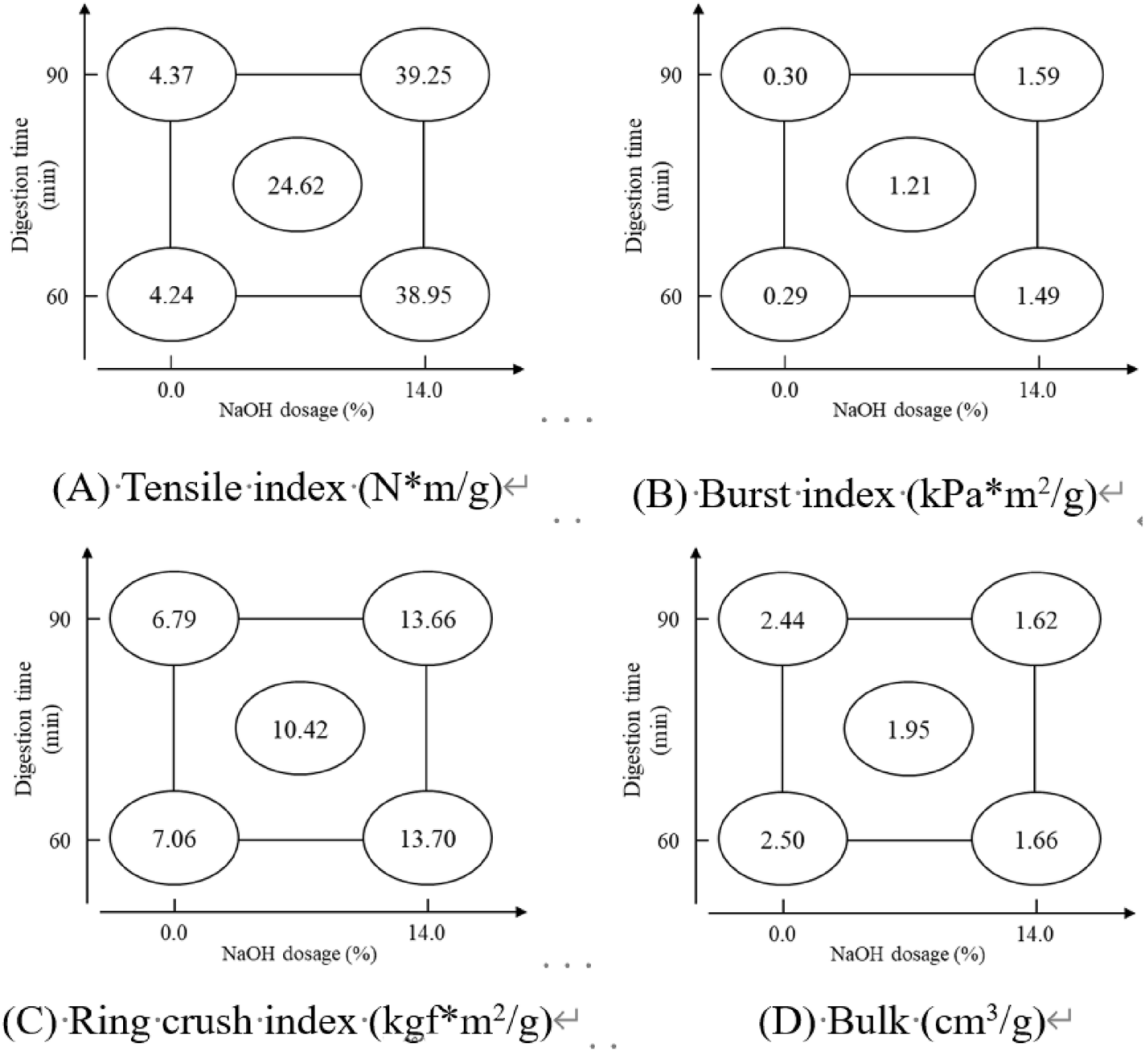
Factorial analysis charts of handsheet properties.

**Figure 9:**
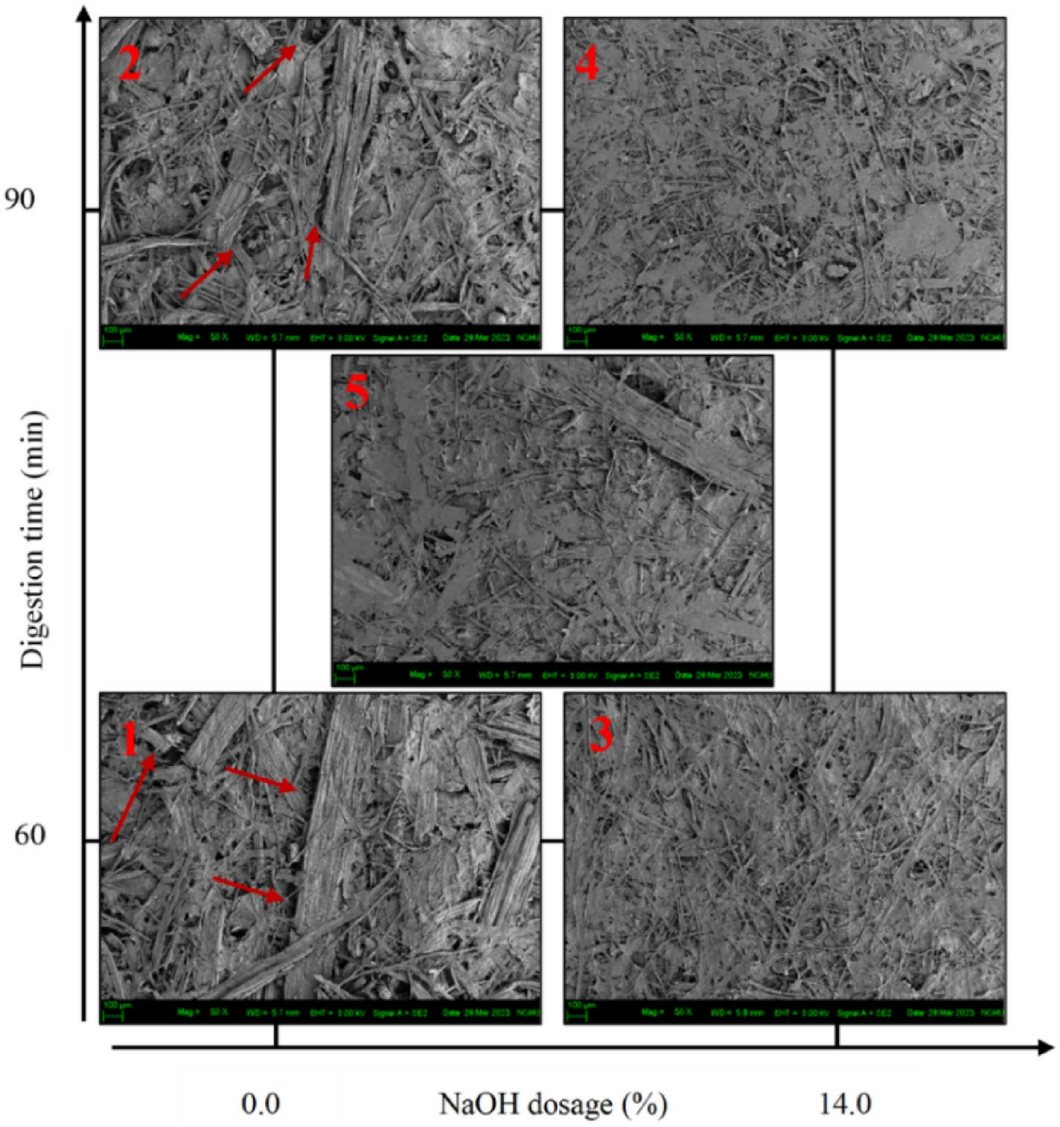
SEM images of the frass handsheets (50X).

Table 6 presents the factorial analysis of handsheet properties. All the properties are significantly influenced by NaOH dosage (X_2_). The strength properties (tensile, burst, and ring crush index) show positive effects, while the bulk is negatively affected. Figure 8 illustrates that increasing the NaOH dosage enhances the strength properties and reduces the bulk. The study also examines handsheet formation using SEM, as shown in Fig. 9. Under a magnifying ratio of 50X, SEM images of factorial points 1 and 2 show that the spacings and holes in the felting structure indicated by red arrows. The structure is denser and more uniform when the NaOH dosage is higher (points 3, 4 and 5). In summary, SEM images of the handsheet evidence the results of physical properties, according to which a tight felting structure exhibits higher strength properties due to increased fiber contact area, resulting in higher bonding strength^46^.

**Table 6:** Factorial analysis of handsheet properties.

Factor	Tensile index (N*m/g)	Burst index (kPa*m^2^/g)	Ring crush index (kgf*m^2^/g)	Bulk (cm^3^/g)
Factor X_1_	0.215	0.052	− 0.154	− 0.045
Factor X_2_	34.794 *	1.244 *	6.752 *	− 0.833 *
X_1_X_2_ interaction	0.092	0.050	0.119	0.014
Assessed value	22.623	0.602	5.238	0.171
Pooled standard deviation (s)	1.780	0.047	0.412	0.013

*Means the factor impact is significant.

*Factor X*_*1*_ Digestion time, *Factor X*_*2*_ NaOH dosage.

To develop the papermaking application of the frass pulp, the study selected the sample with the highest pulping yield (midpoint, 75 min; 7%) and blended the frass pulp sample with TOCC. The mixed pulp handsheet properties are shown in Table 7. As the ratio of frass pulp substitution increases from 25 to 100%, the strength properties and the bulk of the handsheet are enhanced. When the substitution ratio reaches 75%, the burst and ring crush index of the mixed handsheet can attain the strength level of TOCC. Furthermore, at a 100% substitution ratio, the ring crush index can reach the level of JOCC. Simultaneously, the incorporation of frass pulp results in an increase in bulk, surpassing that of TOCC, which just meets the high bulk requirement of the papermaking industry. Furthermore, without the addition of chemical agents, some of the mixed samples can meet the strength quality specifications for B-grade corrugating medium paper and C-grade liner board paper in accordance with the Taiwan CNS standard. In summary, the frass pulp has the potential to substitute for OCC in the production of cardboard.

**Table 7:** Handsheet properties of the frass soda pulp blended with TOCC.

Sample (Blend ratio)	CSF (mL)	Tensile index (N*m/g)	Burst index (kPa*m^2^/g)	Ring crush index (kgf*m^2^/g)	Bulk (cm^3^/g)
100%	245	24.20 ± 0.99 ^b^	1.16 ± 0.07 ^c^	10.40 ± 0.55 ^c^	2.60 ± 0.05 ^c^
75%	295	21.90 ± 1.53 ^b^	1.05 ± 0.04 ^ab^	9.33 ± 0.38 ^b^	1.95 ± 0.03 ^b^
50%	300	21.67 ± 0.90 ^b^	1.11 ± 0.04 ^bc^	9.08 ± 0.29 ^b^	1.95 ± 0.02 ^b^
25%	325	18.73 ± 0.83 ^a^	0.95 ± 0.04 ^a^	7.30 ± 0.16 ^a^	1.93 ± 0.06 ^b^
TOCC	385	28.62 ± 1.70 ^c^	1.03 ± 0.05 ^ab^	9.29 ± 0.04 ^b^	1.79 ± 0.06 ^a^
JOCC	405	32.85 ± 2.063 ^d^	1.37 ± 0.09 ^d^	10.24 ± 0.32 ^c^	1.76 ± 0.03 ^a^
The quality requirement standard for corrugated board paper in CNS					
Corrugating medium (CNS 1455 level B)	–	> 29.43	–	> 7	–
Liner board (CNS 2955 level C)	–	–	2.0	> 10	–

Comparison of the data for different groups was performed by using Turkey’s HSD test. The significant level is set at *p *≤ 0.05; Group a < b < c < d.

## Conclusion

After the cattle dung is treated by BSFL, it can gain the frass that is composite by undigested fiber of cattle dung. The chemical compositions, microstructure and fiber morphology of the frass exhibit potential in pulp and papermaking applications. After the soda pulping factorial experiment, the results show that the pulping properties of the frass are mainly affected by the NaOH dosage. The midpoint condition (75 min, 7%) in the study causes the appropriate disintegration of the fiber, which leads to the highest pulping yield of the experiment. The midpoint pulp sample blend with TOCC can produce a handsheet with a ring crush index that can reach the standard quality requirement of cardboard paper without adding chemical agents. In summary, frass has feasibility in pulp and papermaking applications, especially cardboard production. If the cattle dung is treated by BSFL and the frass can be utilized in multiple fiber applications, the circular utilization value of cattle will be more value-added and have more chance to develop well.

## Data Availability

The datasets presented in this current study are available from the corresponding author on reasonable request.

## Abbreviations

CDF: Cattle dung fiber
BSF: Black soldier fly
OCC: Old corrugated container
TOCC: Taiwan OCC
JOCC: Japan OCC
Fl: Fiber length
Fw: Fiber width
CWT: Cell wall thickness
